# Evidence of promoting prevention and the early detection of breast cancer among women, a hospital-based education and screening interventions in low- and middle-income countries: a systematic review protocol

**DOI:** 10.1186/s13643-018-0889-0

**Published:** 2018-12-14

**Authors:** Adwoa Bemah Bonsu, Busisiwe Purity Ncama

**Affiliations:** 1 Discipline of Nursing, School of Nursing and Public Health, University of KwaZulu-Natal, Durban, 4001 South Africa; 2 Department of Nursing, Kwame Nkrumah University of Science and Technology, Private Mail bag, University Post Office, Kumasi, Ghana

**Keywords:** Women, Breast cancer, Prevention, Early detection, Education, Breast self-examination, Clinical breast-examination, Screening, Hospital-based, Low- and middle-income countries

## Abstract

**Background:**

Given the increasing burden of breast cancer in the low- and middle-income countries, cost-effective approaches are needed to improve the early detection of breast cancer in these continents. Global policies and guidelines are now placing much emphasis on promoting early detection of breast cancer through integrated education and screening interventions. The proposed systematic review aims to map evidence on hospital-based breast cancer education, breast self-examination, and clinical breast examination services for women in low- and middle-income countries.

**Methods/design:**

We will conduct a systematic review of peer-reviewed studies on hospital-based breast cancer prevention intervention (breast cancer education, breast self-examination, and clinical breast examination) for women in low- and middle-income countries. An electronic search will be conducted in the following electronic databases CINAHL Plus with full text (EBSCOhost), MEDLINE with full-text (EBSCOhost) PsychINFO (EBSCOhost), and PubMed. Articles will also be searched through the “Cited by” search and citations included in the reference list of included articles. A two-stage mapping approach will be conducted. The first stage will involve screening studies through assessing their titles. Also, we will screen abstracts of identified studies descriptively and by focus and methods as dictated by the inclusion and exclusion criteria. The second stage will include extraction of data from eligible studies. A parallel screening and data extraction will be conducted by two reviewers. The quality of included studies will be assessed using the mixed methods appraisal tool (MMAT). A narrative account of the data extracted from the included studies will be analyzed using the thematic analysis.

**Discussion:**

We hope to find relevant studies reporting evidence on promoting prevention and the early detection of breast cancer among women in a hospital-based education and screening interventions in low- and middle-income countries. The evidence obtained from the included studies when summarized will help guide future research. The study results will be disseminated electronically and in print. Also, it will be presented at conferences related to breast cancer.

**Systematic review registration:**

The protocol has been registered with PROSPERO, with registration number CRD42017077818.

## Background

Breast cancer (BC) is a malignant growth in any part of the breast and the most common diagnosed cancer among women worldwide [1, 2]. Evidence projects that over 19.5 million new cases of BC will be diagnosed among women globally by the year 2024, of which 55% will occur in low- and middle-income countries (LMICs) [3]. Studies confirm that the majority of women with BC in most LMICs (50–80%) report with metastatic disease due to several factors such as inadequate knowledge on BC and socio-cultural influence on BC [4, 5]; resulting in increasing death rate in these communities. As such, the impact of BC on women, their families and LMICs economy and its resources are substantial, calling for an immediate control and prevention interventions.

Global policies and guidelines suggest prevention as the important priority and the cost-effective approach to curb the world’s burden of BC; especially, in LMICs where inadequate trained oncology health personnel, poor infrastructure, and economical and geographical barriers to BC treatment exist [4, 6–9]. World Health Organization [10] recommends screening and early detection as the two main components of cancer prevention/control for countries with high prevalence and mortality rates of cancer as well as late presentation of most curable cancers [10]. The use of active interventions such as breast cancer education (BCE), breast self-examination (BSE), and clinical breast examination (CBE) has shown to decrease the incidence, late presentation, and death rates of BC among women [11, 12]. Hence, BCE, BSE, and CBE seem the effective prevention/control and early detection measures for BC in limited resource countries [6, 12–14].

CBE and BSE have been strongly endorsed by oncology experts such as the Breast Global Health initiative (BGHI) for LMIC communities due to its contribution to early detection of BC and cost effectiveness [15]. A number of studies suggest that at least BSE improves women literacy and practices on breast in a relatively short time while effective CBE leads to early detection of BC [16–18]. While the benefit of various interventions on BC prevention/control and early detection for women in most developed countries are well documented [11, 12], research that documents such prevention interventions or enhances early detection of BC for women in LMICs appears scant. An intervention is considered as a blend of strategic designed program such as educational program and health promotion campaign intended to alter behavior and influence knowledge, beliefs, and attitudes of individuals or improve health status among individuals or an entire population which may be implemented in varied settings [19]. Though there are numerous BC prevention interventions, in this review, BC prevention intervention will refer to the use of hospital-based BCE, BSE, and CBE services. For this review, BC prevention and early detection interventions will be explored through the practice of BSE among healthy women with no evidence of malignancy and availability of hospital-based BCE, and CBE services for healthy women with no evidence of cancer in LMICs.

The existing literature indicates that there is a need to adapt effective strategy such as BCE and breast screening that could enhance BC prevention and early detection [6, 12–14]. This may significantly reduce incidence and mortality rates associated with BC, facilitate early diagnosis of BC before metastasis, and improve survival of women with BC in LMICs. This is a systematic review protocol. The rationale of this systematic review will therefore be to map evidence of literature on hospital/clinic-based BC prevention and early detection interventions with focus on BCE, BSE, and CBE services for healthy women with no evidence of malignancy in LMICs. The objectives of this systematic review will be as follows:

To review evidence of published literature on existing hospital-based intervention:

- ❖ To raise women awareness regarding risk factors and early warning signs of breast cancer (e.g., lump in breast)
- ❖ On BSE practice among healthy women with undetected cancer but, as yet, do not exhibit any symptoms
- ❖ CBE service for women

The findings from this study will enable the researchers to examine the extent, range, and nature of research activities on hospital-based BC prevention intervention regarding BCE, BSE, and CBE services for women in LMICs. Additionally, the findings will enable the researchers to identify the existing BC prevention models and interventions that improve BC prevention and early detection among women in LMICs.

## Methods/design

### Systematic review

We will carry out a systematic review of peer-reviewed literature on hospital-based BC prevention intervention for women in LMICs. A systematic review method was selected as it enhances the mapping of new concepts, types of evidence and gaps related. For the proposed review, we will be guided by preferred reporting items for systematic review and meta-analysis protocols (PRISMA-P) [20]. The framework includes (i) identifying the research question; (ii) identifying the relevant studies; (iii) selecting of studies; (iv) charting the data; and (v) collating, summarizing, and reporting results.

### Identifying the research question

The main research question is what is known from the existing literature about BC prevention and early detection interventions for women in LMICs? The sub-research questions are as follows:

1. What are the existing hospital/clinic-based models for BC prevention and early detection services in LMICs?
2. What are the available hospital/clinic- based interventions for BCE, BSE and CBE services for women in LMICs?

### Eligibility of research question

The study will use an amended PICOS (Population, Intervention, Comparison, Outcomes and Study setting) framework to determine the eligibility of the research question as shown in Table 1 below.

**Table 1:** PICOS framework for determination of eligibility of review question

Criteria	Determinants
Population	The population of this study will be healthy women with no evidence of malignancy accessing a BCE or screening intervention
Intervention	Patient-focused BCE and raising awareness over risk factors, early BC signs and symptoms, BC screening, and/or detection among women
Comparison	None
Outcomes	• Increased knowledge on BC
	• Practice BSE
	• Access CBE services
	• Early detection of BC
	• Reduced incidence of BC
Study setting	LMICs; within a health care facility (hospital/clinic)

### Identifying relevant studies

Primary studies that have a clear empirical base using qualitative, quantitative, and mixed methods published in peer-reviewed journals as well as in gray literature that address the research question will be included. All study designs will be included. An electronic search will be conducted in the following electronic databases CINAHL Plus with full text (EBSCOhost), MEDLINE with full-text (EBSCOhost) PsychINFO (EBSCOhost) and PubMed. Studies will be identified by searching literature that was published in any language from inception. The search terms will be related to women, BC, health care setting, and prevention/early detection intervention. The search strategy will be modified when necessary to suit syntax requirements. Database-specific thesaurus terms (e.g., MeSH terms) as well as free-text terms will be used to search articles. We will implement our search on databases relevant to LMICs that have been put together in a collaborative effort by Cochrane Groups. To limit our database topic search to studies related to LMICs, a LMICs filter, based on the World Bank classification country list of low-income economies and lower middle-income economies will be employed in proposed databases to retrieve relevant studies. After searching, the studies will be screened against the inclusion and exclusion criteria. Articles will also be searched through the “Cited by” search, and citations included in the reference list of included articles will also be hand searched to identify relevant studies. We used the preferred reporting items for systematic review and meta-analysis protocols (PRISMA-P) as a guide to develop the protocol. Also, the PRISMA-P checklist has been completed to enhance the quality of the protocol [20]. The study protocol has been registered with PROSPERO, with registration number CRD42017077818.

### Study selection

The eligibility criteria were developed to ensure that studies included contain the specific information needed to address the research question on the BC prevention and early detection interventions for women in LMICs.

### Eligibility

#### Inclusion criteria

Studies meeting the following criteria will be included:

- Setting: studies conducted in LMICs within a health care facility. (In this review, health care facility is defined as health care that is based in hospital or clinic).
- Population: healthy women with no evidence of malignancy accessing a BCE or screening intervention.
- All study types will be included.
- Published studies from inception to date.
- Patient-focused BCE and raising awareness over risk factors, early BC signs and symptoms, BC screening, and/or detection among healthy women.
- Outcome: study outcomes will be conceptualized in accordance with the four category frameworks proposed by Harderman and colleagues [23]. This include determinant of behavior (e.g., increased women knowledge), behavioral outcome (e.g., BSE practice and accessing CBE services), physiological and biochemical outcomes (identification of women with pre-malignant disease), and health outcomes (incidence rates and stage of BC presentation).

#### Exclusion criteria

Studies will be excluded if they meet the following characteristics:

- Interventions that solely sought to educate health care professionals on BC awareness or did not specifically include healthy women will be excluded.
- Interventions that solely sought to educate women diagnosed with breast cancer will be excluded.
- Studies that report on community-based interventions will be excluded.
- Studies that will be reported as abstract will be excluded from the review.

The search strategies will be piloted to check the appropriateness of the selected databases and key words. Articles will be searched from the databases by one reviewer who will export all eligible studies into EndNote X7.0.2 reference management software. EndNote X7.0.2 program will be used to check for duplication of articles and to delete the duplicated articles. The reviewer will share the Endnote library with the second reviewer after a comprehensive title screening guided by the criteria for eligibility. Table 2 below illustrates how the electronic data search will be recorded.

**Table 2:** Electronic search record

Date	Keyword search	Search engine used	Number of publication retrieved	Number of publication aftertitle screening
ᅟ	ᅟ	ᅟ	ᅟ	ᅟ

Abstracts and full articles of the included studies will be independently screened for eligibility by two reviewers to identify study analysis and assessment. Where there is no agreement between the two reviewers, the studies will be passed on to a third reviewer for consideration. We will seek for help from the University of KwaZulu-Natal (UKZN) library services for articles that are difficult to access. We will also write to the authors to ask for papers in cases of difficult to find articles. Table 3 below presents the results of the pilot search.

**Table 3:** Results of the pilot database search

Keyword search	Date of search	Search engine used	Number of publications retrieved
(((((“Breast Neoplasms”[Mesh]) AND women) AND ((low and middle income countries)))) AND ((((((((symptoms) OR warning signs) OR detect*) OR diagnos*) OR health promotion) OR prevent*)) OR intervention*)) AND (((((((((((((((((general practitioner*) OR oncologist) OR physician assistant) OR physician assisstant) OR physician) OR clinician) OR doctor) OR nurse assistant) OR OR “midwifery”[All Fields] OR “midwife”[All Fields])) OR midwife) OR nurse) OR breast clinic) OR palliative care clinic) OR oncology clinic) OR clinic) OR health centre) OR hospital)	September 23, 2017	PubMed	31

**Fig. 1:**
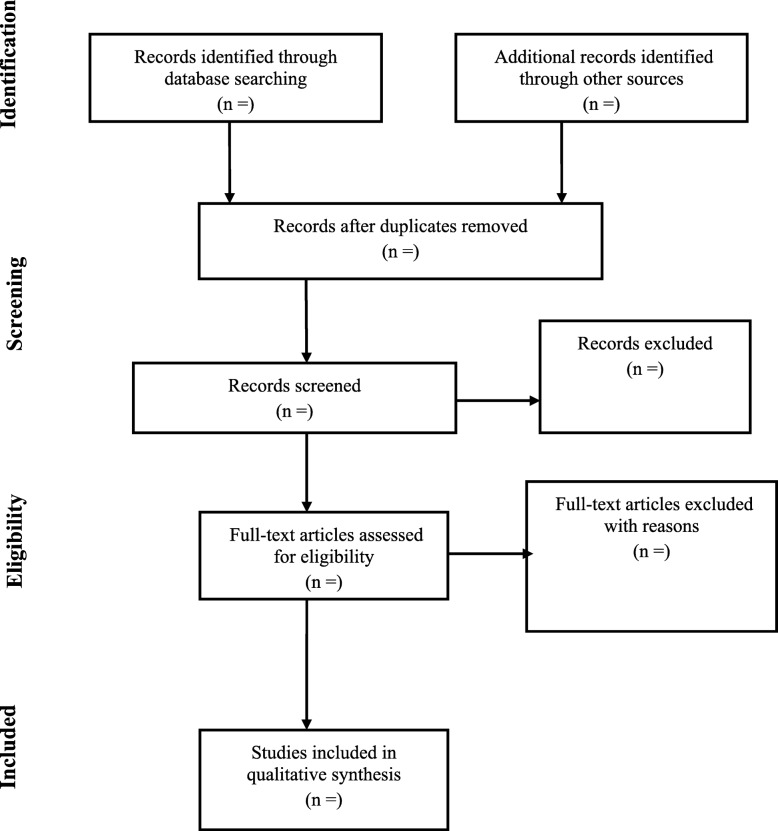
Summary of studies selection procedure using PRISMA

The study selection procedure will also be summarized using a PRISMA chart as indicated in Fig. 1 below.

#### Charting the data

An analytical method will be employed by two reviewers to extract the background information and process-oriented information of each included study. A data charting form will be developed using Google forms. The variables and themes to include in answering the question will be determined as indicated in Table 4. The data charting form will be constantly updated. Differences in data extracted will be resolved through discussion among the reviewers and if consensus is not reach, the project lead will be consulted.

**Table 4:** Data charting (extraction) form

Author and date	
Study title	
Journal full reference	
Study aims	
Study design	
Population characteristics	
Study setting	
Recruitment setting	
Sampling method	
Sample size	
Data collection method	
Data analysis	
Intervention	
Outcome (main findings)	
Significant outcome (most relevant findings)	
Conclusions	
Comments	

#### Collating, summarizing, and reporting of results

The aim of this study is to map the existing evidence and to summarize the findings as presented across studies. A narrative account of the data extracted from the included studies will be analyzed using the thematic analysis. Data will be extracted and described around the following outcomes: determinant of behavior (e.g., increased women knowledge), behavioral outcome (e.g., BSE practice and accessing CBE services), physiological and biochemical outcomes (identification of women with pre-malignant disease), and health outcomes (incidence rates and stage of BC). Emerging themes will also be coded. NVIVO software version 10 will be utilized collectively to code the data from the included studies based on the above categories [21]. The below process will be followed:

- Coding data from the included articles
- Categorizing the codes into major themes
- Displaying the data
- Identifying key patterns in the data and identify sub-themes
- Summarizing

#### Synthesis

We will use the resulting themes and assess their relationship to the research question. The reviewers will also analyze the meanings of the findings relative to the aim of the study and the significance of these results for future research, policy, and practice.

#### Quality appraisal

Quality appraisal is a priority in systematic reviews or part of the systematic review methodology [20]. To enhance the methodological quality of this review, the quality of the studies will be assessed through study appraisal employing the mixed method appraisal tool (MMAT)-Version 2011 [22]. The tool will be used to assess the appropriateness of the aim of the study, adequacy and methodology, study design, participant recruitment, data collection, data analysis, presentation of findings, authors’ discussions, and conclusions. This will help in the determination of the quality of the articles using the aforementioned domains.

## Discussion

The systematic review will be conducted as a first part of the study on the integration of prevention into cancer palliative care: a case study of breast cancer in a tertiary hospital, Ghana. The review is aimed at mapping the existing evidence and summarizing the findings as presented across the studies on promoting prevention and the early detection of breast cancer for women in LMICs. Additionally, the review will identify the existing hospital/clinic-based education and screening models and interventions that enhance access to BCE, BSE, and CBE services for women in LMICs. Despite that there is a growing recognition that health systems should develop and integrate appropriate, cost-effective cancer prevention, and early detection interventions into existing non-communicable disease (NCD) programs especially in LMICs where most curable cancers are initially diagnosed at an advanced stage [6, 8, 12, 23]; there seems to be paucity of evidence on such models or interventions for BC for healthy women in LMICs. In order to reduce the incidence and mortality rate of BC, facilitate early detection of BC among healthy women, and improve survival of women in LMICs, there is a need to explore the BC education and screening services for women, especially in LMICs [8, 23, 24]. Studies that report on education of health care professionals and women diagnosed with BC on BC awareness or did not specifically include healthy women will be excluded because the focus of this review is on healthy women access to BC education and screening services. Also, studies that report on community-based interventions will be excluded because this review is focused on hospital or clinic-based interventions. Deaths in most women could be avoided if BC education and screening services are available and integrated into NCD programs for healthy women who have no evidence of malignancy. This is because physiologically, women are at risk of BC due to certain specific factors [25, 26]. The findings of this study may be of interest to relevant stakeholders especially in LMICs where the burden of BC is significantly high; stakeholders involved in the provision of BC services, as well as stakeholders advocating for the integration of BC prevention and early detection services into existing health care systems. In addition, the findings of this study will be of interest to researchers by highlighting gaps in evidence that may require further investigation.

## Plans to disseminate study results

The study results will be disseminated electronically through publications. Further, the results will be disseminated in print to policy makers and stakeholders involved in the provision of BC services especially in LMICs as well as stakeholders advocating for the integration of BC early detection services into existing health care systems. Also, it will be presented at conferences related to breast cancer.

## Abbreviations

BC: Breast cancer
BCE: Breast cancer education
BGHI: Breast Global Health Initiative
BSE: Breast self-examination
CBE: Clinical breast-examination
LMICs: Low-middle income countries
MMAT: Mixed method appraisal tool
NCD: Non-communicable diseases
PICOS: Population, Intervention, Comparison, Outcome, Study setting
UKZN: University of KwaZulu-Natal
